# Transcriptome analysis of resistance mechanism to potato wart disease

**DOI:** 10.1515/biol-2021-0045

**Published:** 2021-05-10

**Authors:** Peihua Li, Ruihong Fan, Zhengsong Peng, Yuan Qing, Zhirong Fang

**Affiliations:** College of Agronomy, Xichang Univeristy, Xichang, Sichuan 615013, China; College of Agronomy, Sichuan Agricultural University, Chengdu, Sichuan 611130, China

**Keywords:** potato wart disease, food safety, high-throughput sequencing, differentially expressed genes

## Abstract

To understand the molecular mechanism of the resistance to potato wart disease, we used the potato cultivar Qingshu 9 as the experimental material and prepared potato samples with different levels of disease through inoculation. The RNAs of the samples were extracted, and transcriptome analysis was performed on the samples not infected by the disease (control group) and also on the samples with different levels of disease, with the aid of high-throughput sequencing. Next, the differentially expressed genes (DEGs) associated with the resistance to potato wart disease were identified based on the analysis results. Using bioinformatic tools, the DEGs were functionally annotated, classified, and enriched in related metabolic pathways. The main results are as follows: Compared with the control group, 4 DEGs were identified in the samples with light disease, 52 were found in the samples with medium disease, and 214 were discovered in the samples with heavy disease. Potato mainly resists the wart disease by suppressing its gene expression, and the degree of suppression depends on the level of the disease; the disease resistance might be dominated by cellular nucleic acid-binding protein, AP2-like transcription factor, and E3 ubiquitin-protein ligase. This research provides new insights into the molecular mechanism of potato resistance against wart disease.

## 1 Introduction

Hailed as the “second apple” and the “second bread,” potato is the fourth biggest food crop worldwide after wheat, rice, and maize [1]. In China, the planting area of potatoes reaches 5.3333 million hm^2^, only after that of wheat, maize, and rice. The annual output of potatoes in China stands at 100 million tons, more than anywhere else [2]. As China strives to make potato a staple food, the high and stable output of potatoes is increasingly important to food security in the country [3].

Potato wart disease, also called potato canker and black wart of potato, is a devastating soil-borne disease widely distributed in cold, rainy mountain areas [4,5]. The disease is caused by *Synchytrium endobioticum* (Schilb.) Percival, a soil-borne obligate parasitic fungus. Belonging to *Synchytrium*, *Synchytriaceae*, *Chytridiales*, and *Chytridiomycota*, the fungus has no mycelium and cannot be artificially cultivated yet.

The pathogenic fungus, i.e., *S. endobioticum* (Schilb.) Percival, mainly damages the underground parts of potatoes. The fungus causes the host cells to continuously divide, forming large and small cauliflower-shaped tumors and chapped skins on tuber or stolon. The canker tissues are yellowish white in the early stage. With the elapse of time, these tissues become dark brown, soft, and highly putrescible, giving off a foul odor [6,7,8].

Potato cancer was first discovered in Hungary in 1896. With the expansion of planting scale and the inflow of foreign potato varieties, it gradually spread to Central and Northern European countries. It was introduced in North America in 1912 and successively in South America, India, New Zealand, and other places. Now it has spread to about 50 countries on the five continents of the world.

The dormant sporangia of the fungus exist in tubers infected with wart disease and the tiny soil particles on the tuber surface. After the tubers rot, the dormant sporangia are released into the soil. These dormant sporangia can survive in the soil for more than 30 years [9,10,11]. Despite the huge damage caused by the fungus, still no way has been found to effectively prevent *S. endobioticum* (Schilb.) Percival from infecting potatoes, other than breeding for disease resistance.

Two defining features of potato wart disease are serious damage and high-potential threat. On the one hand, the disease directly affects the output and quality of potatoes in the field and causes potatoes to rot in the late harvest and storage period. The inflected potatoes are of poor quality and inedible, resulting in dull sale and major economic losses. On the other hand, it is difficult to remove the pathogenic fungus that contaminates the soil in the short-term. Over the years, various chemical and biological measures have been tried to clean the soil, but to no avail [4].

Therefore, many countries have treated the wart disease as a serious threat and quarantine infected objects in potato production. The disease is characterized by high incidence and fast spread. On average, the output of plots is reduced by 20–30%. In severe cases, the disease could reduce the output by 80% and even cause total crop failure [12]. This calls for in-depth research on the pathogenic mechanism of potato wart disease. The research results help identifying the genes related to the disease, breeding disease-resistant varieties, and ensuring high and stable output and also high-quality potatoes.

In the research work of potato cancer-resistance gene location, Brugmans et al. used the densification of amplified fragment length polymorphism (AFLP) markers and the use of bacterial artificial chromosome (BAC) capture and sequencing technology, and Ballvaora et al. used one highly resistant and two different highly susceptible varieties of tetraploid potatoes to hybridize. Then molecular marker methods such as simple sequence repeat (SSR), inter simple sequence repeat (ISSR), and random amplified polymorphic DNA (RAPD) were used on the F1 population. Obidiegwu et al. used the F1 population obtained by crossing the tetraploid potato high-resistant variety and the high-susceptible variety and used SSR markers and single-nucleotide polymorphism (SNP) chips to analyze. The transcriptome is the sum of all RNAs transcribed by a specific tissue or cell at a developmental stage or functional state, including messenger RNAs (mRNAs), small RNAs, and noncoding RNAs (ncRNAs). Relying on high-throughput sequencing (HTS), transcriptome sequencing determines mRNAs, small RNAs, or ncRNA sequences and quickly obtains the expression level of almost all transcripts of an organ or a tissue of a species under a certain state [13,14]. At present, transcriptome sequencing has been widely applied to major food crops. However, there is no report on its application on potato wart disease.

In this research, the potato cultivar Qingshu 9 was taken as the material. Potato samples with different levels of disease were obtained through inoculation. The samples were subjected to RNA extraction, library construction and sequencing, and preliminary screening of key genes involved in the resistance to the disease. The research results provide new insights into the resistance mechanism of potato wart disease.

## 2 Materials and methods

### 2.1 Materials

Our experiment uses the potato cultivar Qingshu 9 (selection and breeding of Qinghai Academy of Agricultural Sciences, China), which was provided by Sichuan Potato Key Laboratory, Xichang University.

### 2.2 Experimental method

#### 2.2.1 Sample preparation

**Figure 1:**
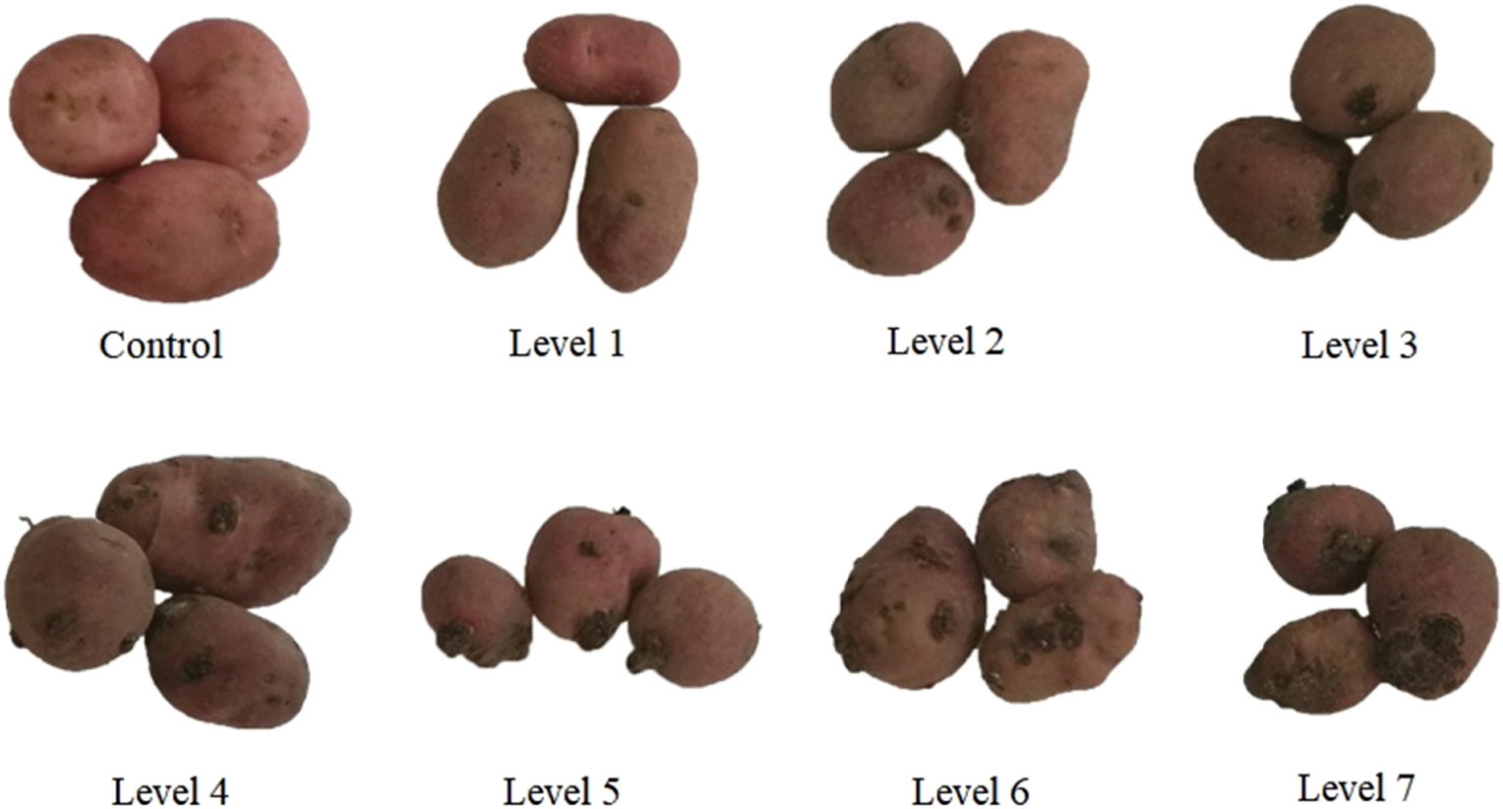
Potato samples with different levels of wart disease.

To identify the differentially expressed genes (DEGs) of the potato infected with the wart disease, three noninfected potatoes were allocated to the control group (L0), and the other potatoes were inoculated with the wart disease of seven different levels (L1–L7) by the Glynne–Lemmerzahl method [6]. The samples with all eight levels of disease are shown in Figure 1.

The inoculation was implemented in the following steps: Apply a 1- to 2-mm-thick warm Vaseline on the eyes of tubers, creating a water-retaining ring. Then drop sterilized distilled water over the eyes. Place disease-causing canker tissues, which has been developed for 3–4 weeks, on the eyes, and cover them up to avoid infection. After incubating at 10 ℃ for 48 h, remove the inoculum, remove Vaseline, and cover the samples with moist sterile soil or peat. Then cultivate the samples at 15–17℃ in the dark for 25–28 days. Observe the growth and cancerization of the samples every other day under the dissecting microscope.

#### 2.2.2 Transcriptome sequences

The total RNAs of potato samples (L0–L7) in Figure 1 were extracted and purified with BioFit plant total RNA purification kit (http://www.biofit.com), where L0 is the control group, L1 and L2 are infected with light disease (light), L4 and L5 are infected with medium disease (medium), and L6 and L7 are infected with heavy disease (heavy).

After passing the test, the RNA samples were sent to BioMarker for library construction and transcriptome analysis by standard methods. The sequencing depth was 6G per sample. The samples in each group were sequenced twice.

#### 2.2.3 Analysis of transcriptome data

The transcriptome data were completely analyzed by CLC Genomics Workbench 10. For the reads of the original sequences obtained through sequencing, the Trim reads program was called to remove the reads with an N (the base information that cannot be determined) proportion greater than 10%, the connectors between the reads of the original sequences, and then the reads with more than half length occupied by bases whose Qpred ≤20. In this way, the reads suitable for transcriptome analysis were obtained.

Next the RNA-Seq analysis program of CLC Genomics Workbench 10 was called to compare the suitable reads with *Solanum tuberosum* (SolTub_3.0) (http://plants.ensembl.org/Solanum_tuberosum/Info/Index), the reference genome of potatoes, revealing the amount of expressed genes under different treatment conditions. The number of expressed genes was estimated by the fragments per kilobase of transcript per million mapped reads; the fold change of the DEGs was expressed in log 2; and the false discovery rate was controlled <0.05.

The DEGs was subjected to Kyoto Encyclopedia of Genes and Genomes (KEGG), Gene Ontology (GO) enrichment analysis, and dynamic GO enrichment analysis, respectively, using KafamKOALA (KEGGs) [15], AgriGO 2.0 [16], and omicshare software (https://www.omicshare.com/).

## 3 Results

Transcriptome analysis showed that seven groups (L0, L1, L2, L4, L5, L6, and L7) were consistent in the number of original reads and the mean length (Table S1). After the unsuitable reads were eliminated by Trim reads program, eight groups had a slight difference in the number of high-quality reads and the mean length. The high-quality reads were located against the reference genome. The results show that the different groups were basically the same in the number of reads that could be identified in the genome. However, both the number of identifiable reads and the number of expressed genes increased with the level of disease.

A total of 214 DEGs were identified through the transcriptome analysis. Only 10 of them witnessed an increase in the number of expressions. Merely four genes were upregulated only under L7 condition (Table S2). For the 204 downregulated genes, 64 genes had log 2 values of less than −2 in at least one of the eight groups.

**Figure 2:**
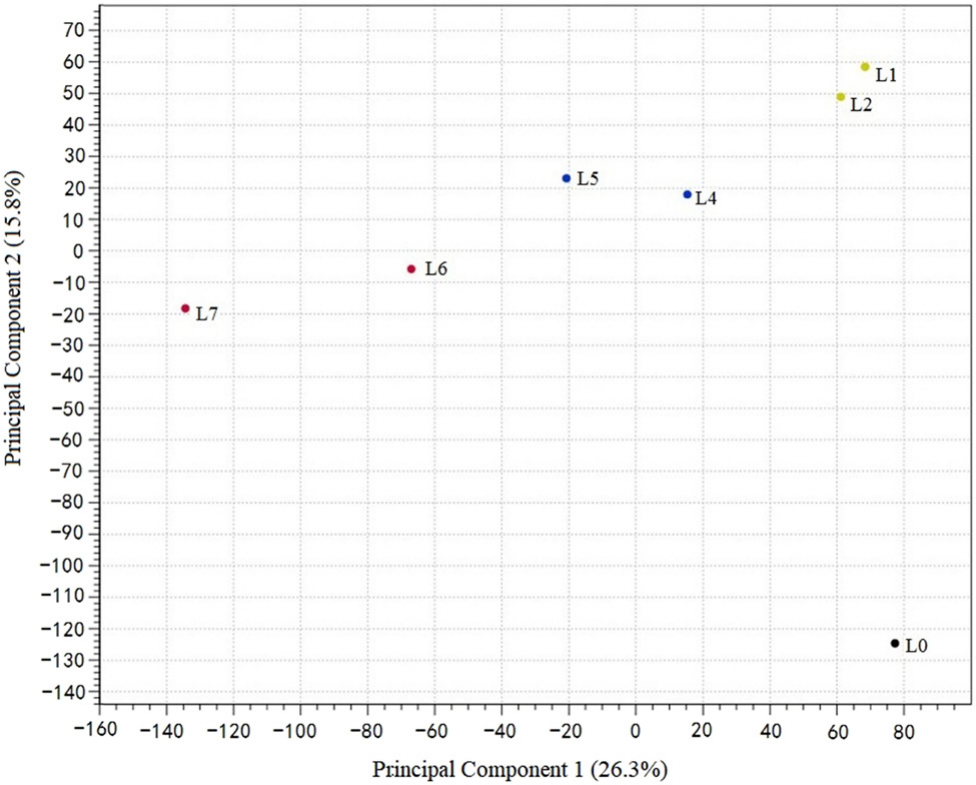
Principal component analysis of the gene expression under different treatments.

Through principal component analysis (PCA), the seven groups (L0, L1, L2, L4, L5, L6, and L7) could be clearly divided into four clusters based on gene expression (Figure 2). L0 is the only group in the lower right cluster, indicating a significant difference between the control samples and infected samples. This proves the success of sample processing. With the growing level of disease, the number of expressed genes gradually shifted to the left, i.e., the gene expression continuously changed as the disease became increasingly severe.

It can also be seen that L1 and L2, L4 and L5, and L6 and L7 form three close clusters, respectively. For convenience, L1 and L2 were defined as light disease cluster (light), L4 and L5 as medium disease cluster (medium), and L6 and L7 as heavy disease cluster (heavy).

Compared with the control group, only 10 genes were downregulated and no gene was upregulated in group L1; 12 genes were downregulated and no gene was upregulated in group L2; 40 genes were downregulated and 3 genes were upregulated in group L4; 74 genes were downregulated and 2 genes were upregulated in group L5; 161 genes were downregulated and 5 genes were upregulated in group L6, that is, 166 genes in this group significantly differed from their counterparts in group L0 in expression; and 204 genes were downregulated and 10 genes were upregulated in group L7, putting the number of DEGs in this group at 214.

To sum up, compared with the control group, the light cluster (a combined analysis of the original data of L1 and L2, rather an addition of the DEGs of L1 and L2) had 4 downregulated genes and no upregulated gene; the medium cluster (a combined analysis of the original data of L4 and L5) had 2 downregulated genes and 3 upregulated genes; the heavy cluster (a combined analysis of the original data of L6 and L7) had 204 downregulated genes and 10 upregulated genes (Table S2). This means the potato mainly responds to the stress of the wart disease through the downregulation of gene expression.

**Figure 3:**
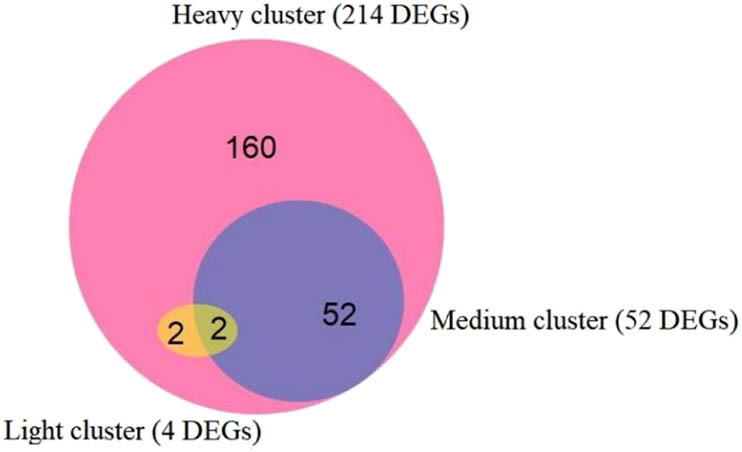
Overlaps of DEGs in three clusters.

Next the overlaps of DEGs in three clusters were analyzed. The results (Figure 3) show that the potato genes were expressed with significant difference between disease conditions. With light disease, the potato samples had no difference from the control group in gene expression. With heavy disease, the potato samples covered the DEGs of those with light and medium diseases. Hence, the gene expression of the potato gradually changes in response to the wart disease.

**Figure 4:**
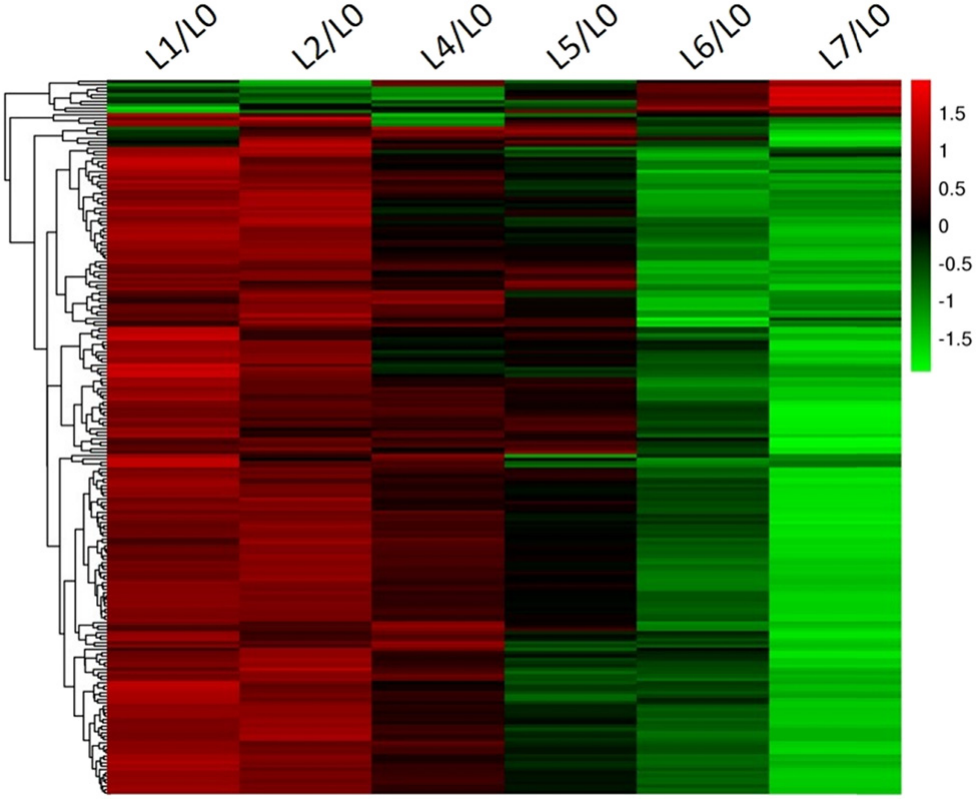
Heat map of the expression of 214 DEGs under six levels of the wart disease.

Figure 4 is the expression trends of 214 DEGs in L1, L2, L4, L5, L6, and L7 obtained through hierarchical clustering. Obviously, the expression of the DEGs either increased or decreased with the growing severity of the wart disease: the expression of most DEGs gradually weakened, while that of a few DEGs gradually enhanced. This further indicates that potato resists the wart disease mainly by suppressing its gene expression.

As shown in Table S3, the DEGs with known functions were distributed on 56 KEGG pathways. Many of these pathways may be closely related to the resistance to potato wart disease. For instance, K09250, the cellular nucleic acid-binding protein, was enriched three times; K09286, an AP2/EREBP transcription factor, was enriched six times; and E3 ubiquitin-protein ligase was enriched three times.

**Figure 5:**
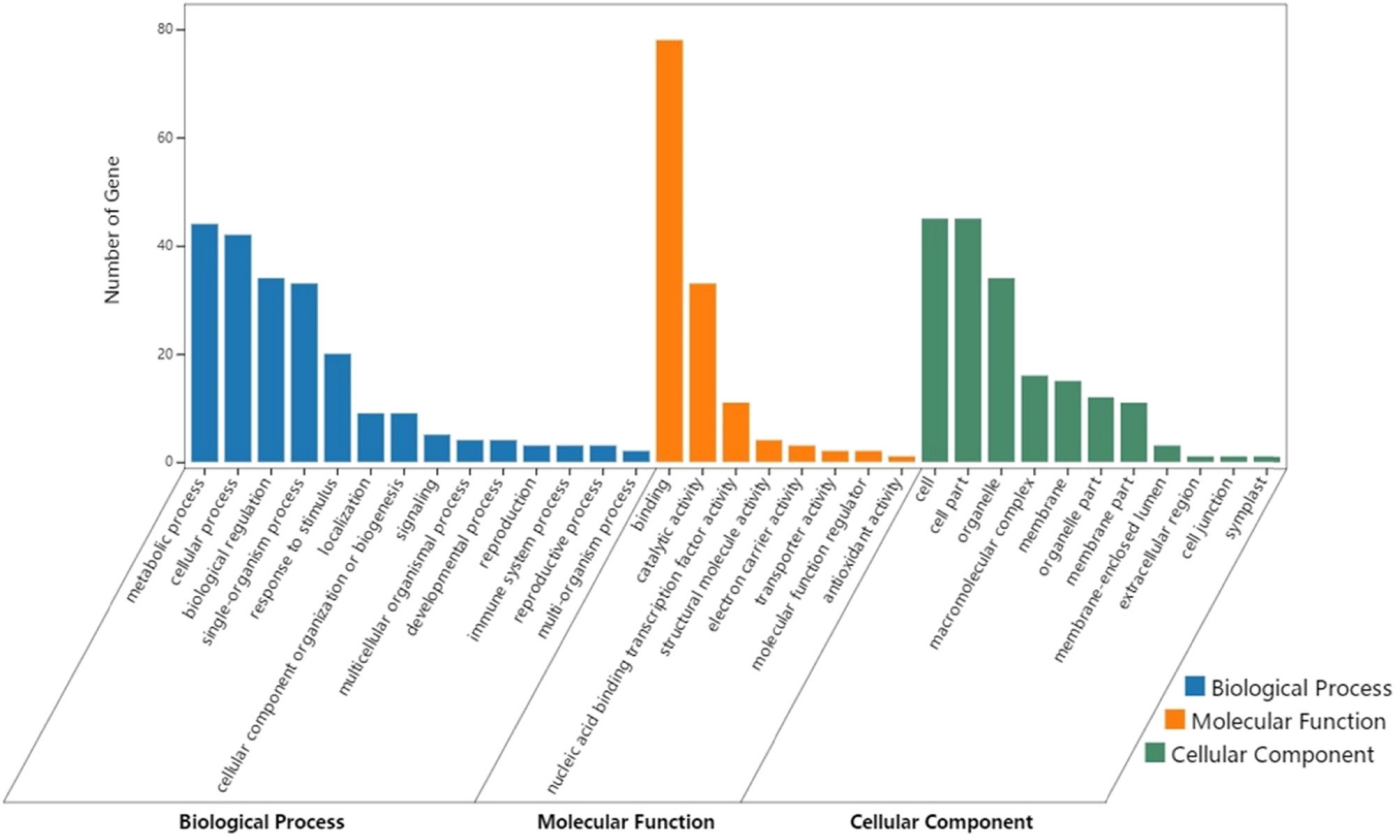
GO classification of the 214 DEGs.

The results of GO enrichment are displayed in Figure 5. In terms of the biological process, the DEGs were heavily enriched in the metabolic process, cellular process, biological regulation, single-organism process, and response to stimulus. In terms of the molecular function, the DEGs were abundantly enriched in binding, catalytic activity, and nucleic acid-binding transcription factor activity. In terms of cellular components, the DEGs were significantly enriched in cells, cell parts, organelles, macromolecular complexes, and membranes.

## 4 Discussion

With serious damage and high-potential threat, the wart disease is a major ailment of potato. Southwestern China used to suffer heavy loss of potato production due to the disease, which severely restricts the growth, output, and quality of the crop. Depending on the level of disease, potato will undergo changes in phenotype, physiology, biochemistry, and molecules. In the previous studies, except for the Sen1 locus on the long arm of chromosome XI, which was recognized by researchers, there were obvious differences in other locus; and so far, these molecular markers have not accurately been located in genes (sequences). Many of these changes are the result of the regulation of the complex inherent molecular network. Therefore, this article screens and identifies the DEGs through bioinformatics analysis, aiming to disclose the potato wart disease resistance mechanism.

With the aid of the HTS, potato samples with different levels of disease were subjected to transcriptome sequencing. Through the PCA and DEGs distribution analysis, the authors learned that the samples with different levels of disease greatly differed from those not infected with the disease. Based on the PCA results on gene expression (Figure 2), L1 and L2 were assigned to the light cluster, L4 and L5 to the medium cluster, and L6 and L7 to the heavy cluster. Compared with the control group, the downregulated DEGs were 3 times that of the upregulated DEGs in the light cluster; the downregulated DEGs were 20 times that of the upregulated DEGs in the medium cluster and the heavy cluster; 198 DEGs were detected in the heavy cluster.

After that, the DEGs were annotated in the KEGG and GO databases. Only a few DEGs could be found in these databases. This result is common in many plants that have been sequenced [17]. A possible reason is that many nonmodel plants have not been sequenced, failing to provide the relevant research data. These unannotated genes should be further analyzed to determine their role in plant growth and enrich the gene databases.

According to our research results, after GO functional annotation and KEGG pathway analysis, the DEGs were found to mainly enrich in the biosynthesis of secondary metabolites of metabolic pathway, biosynthesis of amino acids, intercellular signaling, negative regulation of enzyme activity, and hormone response. These pathways might promote the response to potato wart disease.

The molecular response of potato disease resistance is a complex process. Besides metabolite synthesis and the regulation of disease-resistant cell-signaling pathways, the immune response induced by elicitors also plays an important role, including the nucleotide-binding site (*NBS*)–leucine-rich repeat (*LRR*) genes [18]. The *NBS-LRR* are a family of disease-resistant genes widely present in plants. However, our transcriptome analysis did not find any differentially expressed *NBS-LRR* genes. This highlights the complexity of potato resistance to wart disease and the difficulty in breeding wart-resistant potatoes.

Transcriptome sequencing is a nondestructive and efficient technique to process many genes. This technique has been extensively applied to optimize gene organization, discover unknown genes, and analyze differential gene expression. Many crops have been studied through transcriptome sequencing, such as wheat [17], rapeseed [19], *Brachypodium* [14], and sunflower [20]. This article is the first to apply transcriptome sequencing to analyze the molecular response mechanism of potato wart disease. Our efforts expand the understanding of the complex mechanism of crop disease resistance.

The future research needs to systematically analyze the resistance components of the potato to the wart disease, especially the DEGs critical to disease resistance. The systematic analysis will lay a theoretical basis for the breeding of disease-resistant potatoes and the prevention of potato wart disease.

## 5 Conclusion

This article carries out transcriptome sequencing of potatoes with different levels of disease, revealing that the potato mainly resists the wart disease by downregulating its gene expression. In addition, the DEGs were found to enrich pathways like cell metabolism, cell cycle, biosynthesis, and enzyme catalysis. This means the response to potato wart disease might be dominated by such pathways as bioregulation, chemical reaction, abiotic stimulus response, oxygenated compound response, organic matter response, etc. The research results lay the theoretical basis for screening disease-resistant genes and breeding disease-resistant potatoes.
